# Responses of Hybrid Rice (*Oryza sativa* L.) Plants to Different Application Modes of Nanosized Selenium

**DOI:** 10.3390/plants13223179

**Published:** 2024-11-13

**Authors:** Qianqian Zhang, Haowen Luo, Pipeng Xing, Qichang Gu, Wentao Yi, Xianghai Yu, Changjian Zuo, Xiangru Tang

**Affiliations:** 1 State Key Laboratory for Conservation and Utilization of Subtropical Agricultural Bioresources, College of Agriculture, South China Agricultural University, Guangzhou 510642, China; 2 Scientific Observing and Experimental Station of Crop Cultivation in South China, Ministry of Agriculture, Guangzhou 510642, China; 3 Guangzhou Key Laboratory for Science and Technology of Aromatic Rice, Guangzhou 510642, China; 4 Green Huinong Biotechnology (Shenzhen) Co., Ltd., Shenzhen 518107, China

**Keywords:** antioxidants, plant growth, nanometer materials, hybrid rice (*Oryza sativa* L.), selenium

## Abstract

The application of selenium (Se) fertilizer not only promotes crop growth but also meets the human demand for Se by increasing the Se content in food. However, the application of nanosized selenium (nano-Se) in hybrid rice (*Oryza sativa* L.) production has not been reported. Therefore, the present study conducted a field experiment to investigate hybrid rice’s performance under the different application modes of nano-Se. The nano-Se solution was foliar-applied: once at the end of the tillering (S1), heading (S2), and grain-filling (S3) stages or twice at the end of the tillering stage and the heading stage (S4), and at the end of the tillering stage and the grain-filling stage (S5). The treatment without Se application was taken as the control (CK). The results showed that compared with CK, the S1, S2, S3, S4, and S5 treatments increased the grain yield by 27.83–40.60%, 16.06–25.95%, 14.78–40.86%, 20.94–43.79%, and 22.41–43.52%, respectively. The highest or equally highest grain yield was recorded in the S1 treatment. Yield-related traits including the effective panicle number, grain number per panicle, seed-setting rate, and 1000-grain weight significantly increased under nano-Se treatments. Compared with CK, nano-Se treatment increased the SPAD value (chlorophyll content), net photosynthetic rate, and dry matter accumulation by 3.82–32.83%, 2.85–59.55%, and 8.09–55.29%, respectively. An 11.51–572.85% higher grain Se content was recorded in nano-Se treatments than CK. Moreover, nano-Se application significantly enhanced the activity of superoxide dismutase and catalase. In conclusion, the foliar application of nano-Se enhanced the growth and yield formation of hybrid rice plants, and the S1 treatment was considered as the best application due to having the highest yield.

## 1. Introduction

Rice (*Oryza sativa* L.) is a major cereal in the world and feeds more than half of the world’s population. For half a century, population expansion and dietary structure shifts have led to changes in global grain demand [1]. Due to the population expansion in China, the transformation of dietary structure has triggered an increase in grain demand, and the requirement for rice is expected to keep increasing in the coming years [2]. Therefore, it is important to increase rice yield and improve the stability of rice to ensure world food security.

Hybrid rice was first launched by the Ministry of Agriculture in China in 1996 [3]. As a significant achievement of Chinese agricultural technology, the yield of hybrid rice is 15–20% higher than traditional rice varieties because of heterosis [3,4]. The emergence and promotion of hybrid rice have to some extent fulfilled the growing demand for food among the world population. A previous report indicated that the hybrid rice variety out-yields inbred rice varieties by about 10% which is important to China’s food security [5]. However, despite the higher yield of hybrid rice, its yield potential has not been fully developed and more improved technologies such as fertilization, water management, and plant growth regulators are required to improve the yield formation of hybrid rice varieties [6,7].

Microelement fertilization is a common practice to improve crop productivity in agriculture production. Selenium (Se) is a beneficial nutrient element for plants [8]. Many studies have shown that Se application could not only improve the growth and development of crops but also enhance Se fortification. For example, the study of Gao F et al. [8] demonstrated that the application of Se fertilizer substantially improved the agronomic traits and yield of dryland maize. The study of Yuan et al. [9] showed that spraying Se solution at the full heading stage of rice plants can increase the grain Se content by 91.57%. An early study showed that Se application not only enhanced yield formation but also improved some grain quality parameters of rice [10]. Nanosized selenium (nano-Se) is a new kind of nanomaterial and also a new source of Se fertilizer, which is a red and simple substance Se with higher biological activity and lower toxicity than traditional Se. However, the application of nano-Se in hybrid rice production has not been reported and the effects of nano-Se application on the growth and development of hybrid rice plants are unknown. Hence, revealing the effects of nano-Se on hybrid rice performance and amending the application methods of nano-Se in rice production will not only improve the promotion of nano-Se application in agriculture production but also help to achieve the goal of higher rice yields.

The present study conducted a field experiment and aimed to investigate the effects of the foliar application of nano-Se at different stages on hybrid rice performance. We hypothesized that the foliar application of nano-Se could improve the growth of hybrid rice plants and lead to an increment in grain yield. This study will provide more insights into hybrid rice cultivation, as well as Se application in crop production.

## 2. Results

### 2.1. Effects of Nano-Se on the Yield and Yield-Related Traits of Hybrid Rice

The foliar application of nano-Se substantially improved the yield formation of hybrid rice (Table 1). Variance analysis showed that nano-Se treatments significantly impacted the grain yield, effective panicle number, seed setting rate, and 1000-grain weight. Compared with CK, the S1, S2, S3, S4, and S5 treatments increased the grain yield by 27.83–40.60%, 16.06–25.95%, 14.78–40.86%, 20.94–43.79%, and 22.41–43.52%, respectively. The highest or equally highest grain yield was recorded in the S1 treatment in both seasons and for both cultivars. A higher effective panicle number per plant was recorded in the S1 treatments than CK in both seasons and for both cultivars. There was no significant difference between CK and nano-Se treatments in grain number per panicle except the S1 treatment for Jingnongsimiao in the early cropping season, which was 27.54% higher than CK. Compared with CK, the S4 treatment significantly increased the seed-setting rate of Jingnongsimiao by 23.75% and 19.96% in the early and late cropping seasons, respectively. A 9.64% and 6.53% higher 1000-grain weight was recorded in the S4 treatment than CK for Wufengyou615 in the early and late cropping seasons, respectively.

### 2.2. Effects of Nano-Se on the Grain Quality Parameters of Hybrid Rice

As shown in Table 2, there was no significant difference between the CK and nano-Se treatments in the brown rice rate, milled rice rate, and chalkiness degree for either cultivar in either cropping season. Variance analysis showed that nano-Se treatments significantly impacted the brown rice rate and milled rice rate. A higher head rice rate was recorded in the S1 treatment than in CK for Wufengyou615 in the early season. Compared with CK, Se treatment significantly increased the head rice rate for both cultivars in the late season. There was no significant difference between CK and nano-Se treatments in chalky rice rate for either cultivar in the early season. A higher chalky rice rate was recorded in the S2 and S5 treatments than in CK for Jingnongsimiao in the late season.

### 2.3. Effects of Nano-Se on the Grain Se Content of Hybrid Rice

**Figure 1:**
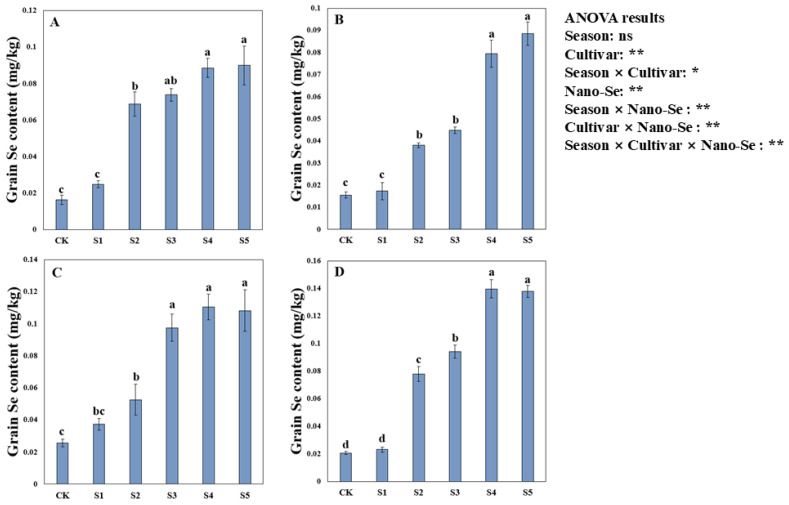
Effects of nano-Se on the grain Se content of hybrid rice: (**A**) for Jingnongsimiao in the early season; (**B**) for Jingnongsimiao in the late season; (**C**) for Wufengyou615 in the early season; (**D**) for Wufengyou615 in the late season. Bars sharing a common letter do not differ significantly at *p* ≤ 0.05 in each cropping season according to the LSD test. For ANOVA results, * and ** represent a significant difference at *p* < 0.05 and *p* < 0.01, respectively; ns represents a non-significant. The nano-Se solution was foliar-applied: once at the end of the tillering (S1), heading (S2), and grain-filling (S3) stages or twice at the end of the tillering stage and the heading stage (S4), and at the end of the tillering stage and the grain-filling stage (S5). The treatment without Se application was taken as the control (CK), the same as below.

Foliar application of nano-Se remarkably improved the grain Se content in hybrid rice (Figure 1). Variance analysis showed that nano-Se treatments significantly impacted the grain Se content of hybrid rice and the Se content significantly differed between the two cultivars. For Jingnongsimiao in the early season, 317.32%, 348.24%, 438.05%, and 446.32% higher grain Se contents were recorded in the S2, S3, S4, and S5 treatments than in CK. For Jingnongsimiao in the late season, 105.25%, 280.46%, 331.03%, and 322.27% higher grain Se contents were recorded in the S2, S3, S4, and S5 treatments than in CK. Moreover, compared with CK, the S2, S3, S4, and S5 treatments also significantly increased the grain Se content of Wufengyou615 in both cropping seasons.

### 2.4. Effects of Nano-Se on the Chlorophyll Content of Hybrid Rice

**Figure 2:**
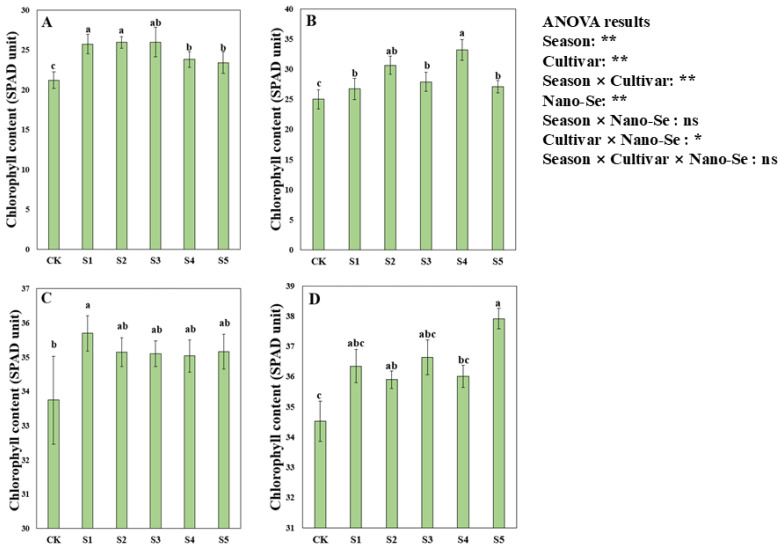
Effects of nano-Se on chlorophyll content in the SPAD unit of hybrid rice: (**A**) for Jingnongsimiao in the early season; (**B**) for Jingnongsimiao in the late season; (**C**) for Wufengyou615 in the early season; (**D**) for Wufengyou615 in the late season.

Foliar application of nano-Se significantly increased the chlorophyll content in terms of the SPAD value of hybrid rice (Figure 2). Variance analysis showed that nano-Se treatments significantly impacted the SPAD value of hybrid rice, and the SPAD value significantly differed due to different cultivars and cropping seasons. For Jingnongsimiao in the early season, compared with CK, the S1, S2, and S3 treatments significantly increased the SPAD value by 21.42%, 22.36%, and 22.59%, respectively. For Jingnongsimiao in the late season, 22.63% and 32.83% higher SPAD values were recorded in S2 and S4 treatments than in CK. For Wufengyou615 in the early season, a 5.78% higher SPAD value was recorded in the S1 treatment than in CK. For Wufengyou615 in the late season, compared with CK, the S2 and S5 treatments significantly increased the SPAD value by 4.00% and 9.85%, respectively.

### 2.5. Effects of Nano-Se on the Net Photosynthetic Rate of Hybrid Rice

**Figure 3:**
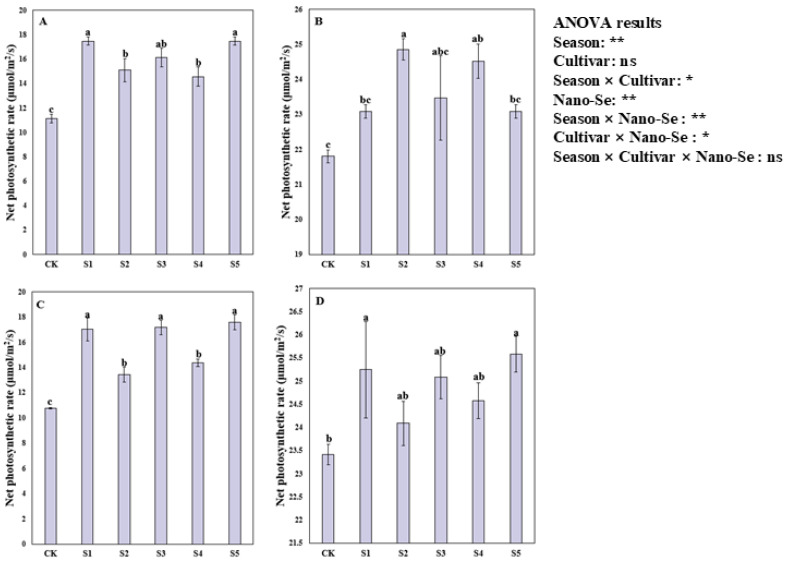
Effects of nano-Se on the net photosynthetic rate of hybrid rice: (**A**) for Jingnongsimiao in the early season; (**B**) for Jingnongsimiao in the late season; (**C**) for Wufengyou615 in the early season; (**D**) for Wufengyou615 in the late season.

Foliar application of nano-Se significantly increased the net photosynthetic rate of hybrid rice (Figure 3). Variance analysis showed that nano-Se treatments significantly impacted the net photosynthetic rate of hybrid rice and the net photosynthetic rate significantly differed between the two cropping seasons. For Jingnongsimiao in the early season, the S1, S2, S3, S4, and S5 treatments significantly increased the net photosynthetic rate by 57.01%, 35.59%, 44.91%, 30.94%, and 57.01% compared with CK. For Jingnongsimiao in the late season, 13.97% and 12.47% higher SPAD values were recorded in the S2 and S4 treatments than in CK. For Wufengyou615 in the early season, compared with CK, the S1, S2, S3, S4, and S5 treatments significantly increased the net photosynthetic rate by 58.06%, 24.63%, 59.55%, 33.43%, and 63.33%, respectively. For Wufengyou615 in the late season, a 7.80% and 9.22% higher net photosynthetic rate was recorded in the S1 and S5 treatments than in CK.

### 2.6. Effects of Nano-Se on the Dry Matter Accumulation of Hybrid Rice

**Figure 4:**
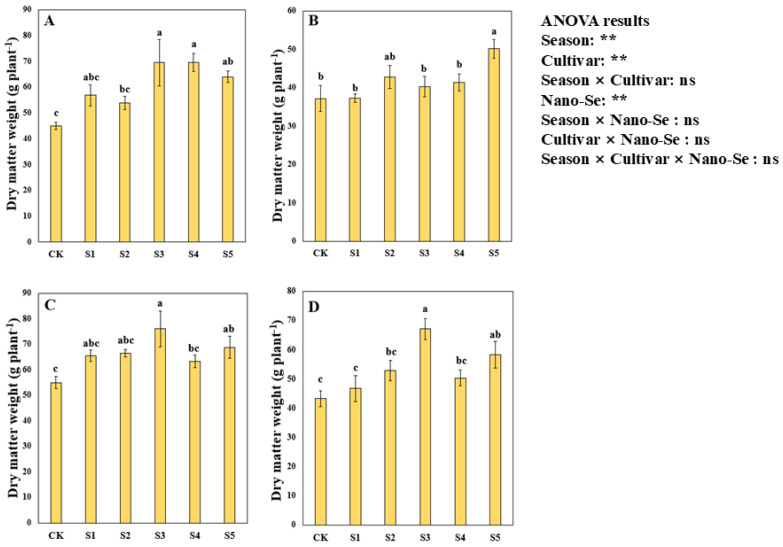
Effects of nano-Se on the dry matter weight of hybrid rice: (**A**) for Jingnongsimiao in the early season; (**B**) for Jingnongsimiao in the late season; (**C**) for Wufengyou615 in the early season; (**D**) for Wufengyou615 in the late season.

Nano-Se application substantially enhanced the dry matter accumulation of hybrid rice (Figure 4). Variance analysis showed that nano-Se treatments significantly impacted the dry matter accumulation of hybrid rice, and the dry matter accumulation significantly differed due to different cultivars and cropping seasons. For Jingnongsimiao in the early season, the S1, S2, S3, S4, and S5 treatments significantly increased the dry matter weight by 26.24%, 19.85%, 54.37%, 54.46%, and 42.18% compared with CK. For Jingnongsimiao in the late season, a 34.77% higher dry matter weight was recorded in the S5 treatment than in CK. For Wufengyou615 in the early and late seasons, a higher dry matter weight was recorded in the S3 and S5 treatments than in CK.

### 2.7. Effects of Nano-Se on the Antioxidant Enzymatic Activity of Hybrid Rice

**Figure 5:**
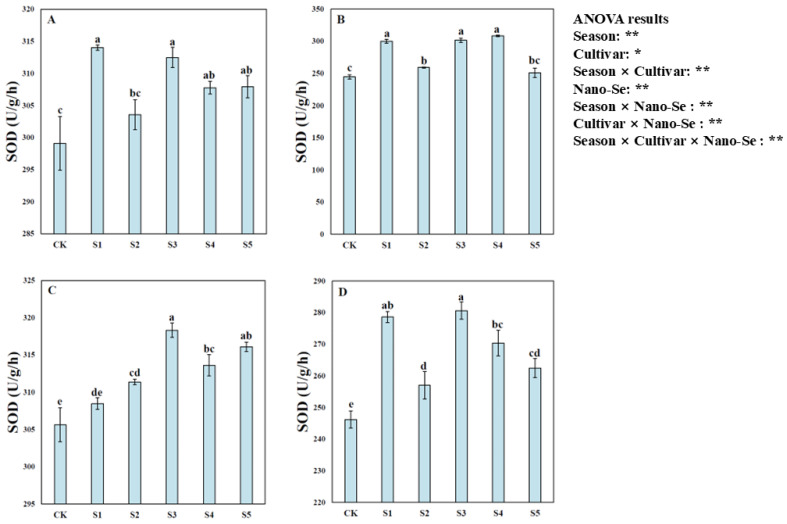
Effects of nano-Se on the SOD activity of hybrid rice: (**A**) for Jingnongsimiao in the early season; (**B**) for Jingnongsimiao in the late season; (**C**) for Wufengyou615 in the early season; (**D**) for Wufengyou615 in the late season.

**Figure 6:**
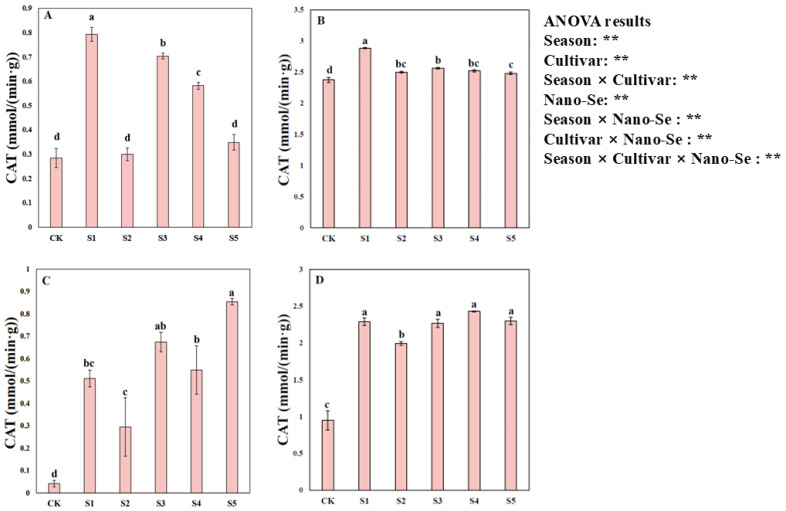
Effects of nano-Se on the CAT activity of hybrid rice: (**A**) for Jingnongsimiao in the early season; (**B**) for Jingnongsimiao in the late season; (**C**) for Wufengyou615 in the early season; (**D**) for Wufengyou615 in the late season.

Nano-Se application substantially enhanced the activity of SOD and CAT in hybrid rice (Figure 5 and Figure 6). Variance analysis showed that nano-Se treatments significantly impacted the activity of SOD and CAT in hybrid rice and the activity significantly differed due to different cultivars and cropping seasons. Compared with CK, the S1 and S2 treatments significantly increased SOD activity for Jingnongsimiao in both cropping seasons. A higher SOD activity was recorded in the S3, S4, and S5 treatments than in CK for Wufengyou615 in both cropping seasons. Moreover, compared with CK, the S1, S3 and S4 treatments significantly enhanced CAT activity for both cultivars in both cropping seasons.

### 2.8. Correlation Among the Grain Yield, Yield-Related Traits, Chlorophyll Content, Net Photosynthetic Rate, Dry Matter Accumulation, and Antioxidants

**Figure 7:**
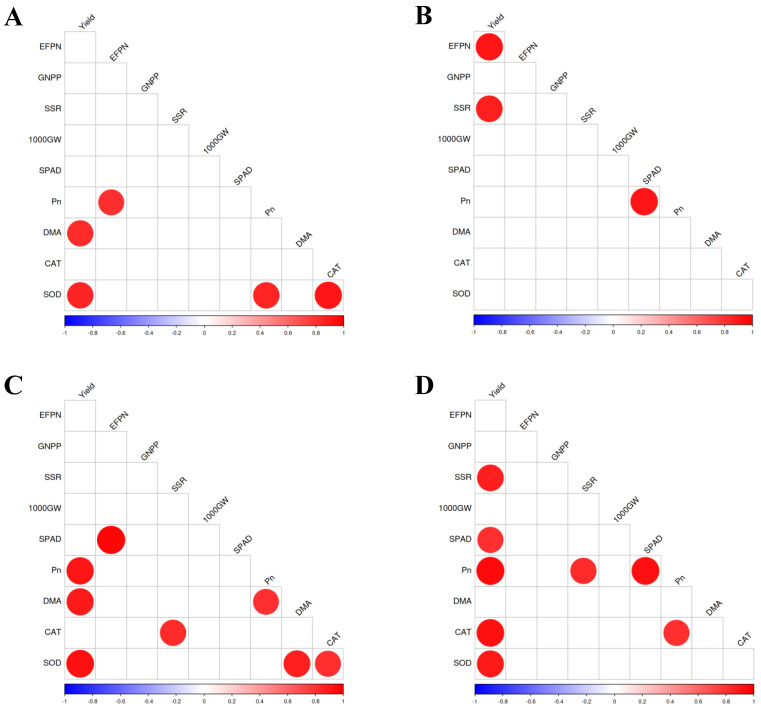
Correlation among grain yield, yield-related traits, chlorophyll content, net photosynthetic rate, dry matter accumulation, and antioxidants: (**A**) for Jingnongsimiao in the early season; (**B**) for Jingnongsimiao in the late season; (**C**) for Wufengyou615 in the early season; (**D**) for Wufengyou615 in the late season. The marked points mean *p* < 0.05. EFPN is the effective panicle number; GNPP is the grain number per panicle; SSR is the seed-setting rate; 1000 GW is the 1000-grain weight; Pn is the net photosynthetic rate; DMA is the dry matter accumulation.

As shown in Figure 7, there were some significant correlations among the measured parameters. For Jingnongsimiao in the early season, yield was significantly and positively correlated with both dry matter accumulation and SOD activity. For Jingnongsimiao in the late season, there was a significant and positive correlation between the SPAD value and the net photosynthetic rate, while yield was significantly and positively correlated with both the effective panicle number and the seed-setting rate. For Wufengyou615 in the early season, yield was significantly and positively correlated with the net photosynthetic rate, dry matter accumulation, and SOD activity. For Wufengyou615 in the late season, yield was significantly and positively correlated with the seed-setting rate, SPAD value, net photosynthetic rate, CAT activity, and SOD activity.

## 3. Discussion

The present study revealed the effects of nano-Se application on hybrid rice performance in terms of growth, yield formation, grain quality, and grain Se content. The results of this study agreed with our hypothesis that the foliar application of nano-Se enhanced the growth of hybrid rice plants and led to an improvement in grain yield. Our findings were consistent with our latest study about nano-Se on aromatic rice cultivars which also showed that nano-Se substantially increased the grain yield [11]. The increment in yield formation might be explained by the enhanced photosynthesis. In our study, the foliar application of nano-Se substantially increased chlorophyll, the net photosynthetic rate, and dry matter accumulation. An adequate matter accumulation through photosynthesis is the basis for achieving a high yield in rice plants. Our results agreed with the study by Yan et al. [12] which indicated that Se fertilizer could significantly enhance the growth and development of crops. The study by Ei et al. [13] also showed that Se application could improve plant growth and development and increase dry matter accumulation. In our study, the improvement of the net photosynthetic rate due to Se application might be attributed to the increased chlorophyll content. There is a close relationship between the chlorophyll content and the net photosynthetic rate in plants. A previous study also indicated that changes in chlorophyll content impacted the yield formation of rice plants through photosynthesis [14].

In addition, we observed that foliar application of nano-Se significantly enhanced antioxidant enzymatic activity in terms of SOD and CAT. Our results were consistent with our previous study which showed that nano-Se application enhanced the activity of antioxidant enzymes in rice plants under cadmium stress [15]. SOD and CAT are important enzymes in defending against oxidative stress in plants, and the enhancement of SOD and CAT activity would be beneficial for improving stress resistance. We deduced that the enhanced activity of SOD and CAT could be one of the reasons for increased yield considering rice plants grow for a long time in paddy fields and the microclimate in paddy fields could be complicated and result in short-term oxidative stress to rice plants [16], while enhancing the activity of SOD and CAT could ensure the stability of yield formation by alleviating the oxidative stresses. The results of our study also showed significant and positive correlations between yield and the activity of CAT and SOD.

Se is an essential nutrient element for humans which is thought to protect against cancer, and Se fortification in food would be beneficial to human health [17]. In this study, we observed a higher grain Se content under nano-Se treatments which indicated that the application of nano-Se can achieve the goal of Se fortification in rice. Our findings were similar to the study of Yan et al. [18], which showed that Se application increased the grain Se content in oats (*Avena sativa* L.) and rice, respectively. Furthermore, we observed that the grain Se content in the rice reached 271.2–306.5 mu g/kg after nano-Se application, which met the standard of Se-rich rice [19]. In addition, we observed that there was no significant difference between the S1 treatment and CK in grain Se content which might be explained by the fact that the nano-Se content was applied in the middle stage of growth of the rice in the S1 treatment and thus the Se accumulated in the leaf and stem and was not translocated to the grain. This finding indicated that Se application should be applied at the late stages (heading stage and grain-filling stage) of rice growth for the purpose of increasing the grain Se content. However, the mechanisms of absorption and translocation of Se in rice plants remain unknown which requires more studies to be conducted at the physiological and molecular levels.

In addition, the present study compared the effects of nano-Se applied in different modes and the best application was applying nano-Se once at the end of tillering, considering the highest yield. However, other forms of Se including sodium selenate and sodium selenite supply Se to crops in the form of ions whilst nano-Se is a simple substance Se, and thus there might be some differences in the absorption and translocation mechanisms between nano-Se and other kinds of Se. The limitation of this study is that we did not explore the differences between nano-Se and other forms of Se on rice performance. Therefore, more studies should be conducted to explore the effects of different forms of Se on the growth and development of rice plants.

## 4. Materials and Methods

### 4.1. Plant Materials and Crop Management

**Figure 8:**
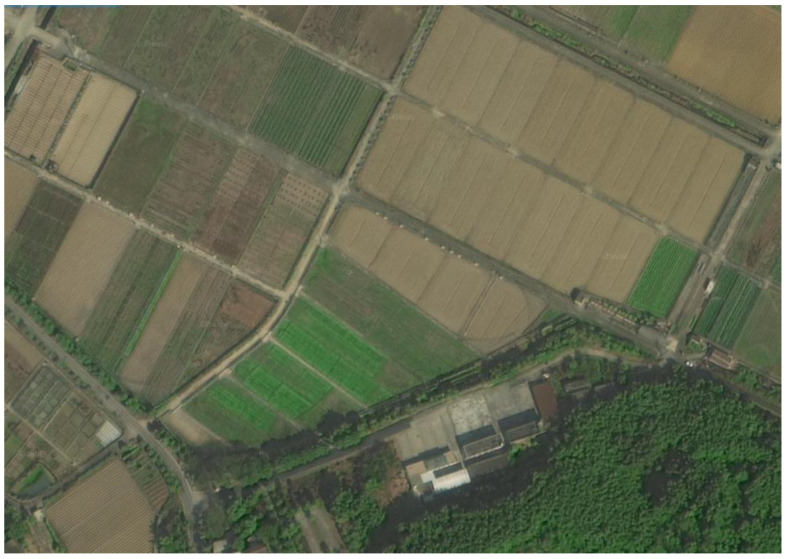
The site of the field experiment.

A field experiment with two cropping seasons was conducted at the Teaching and Research Base, South China Agricultural University (23°14′14 N, 113°38′14 E), Guangzhou City, China, in 2023 (Figure 8). The experimental soil was sandy loam with 19.55 g kg^−1^ organic matter, 1.19 g kg^−1^ total nitrogen, 23.47 μg kg^−1^ Se, and 5.31 pH. Two hybrid rice cultivars, Wufengyou615 (WufengA × Guanghui615) and Jingnongsimiao (Jinhuaruanzhan × Guinongzhan), which have been widely planted in South China for rice production, were used as plant materials. In the early cropping seasons, the sowing and transplanting were carried out in April and the harvest was carried out in July. In the late cropping seasons, the sowing and transplanting were carried out in July and the harvest was carried out in November. A commercial fertilizer manufactured by Dongguan Foota, Ltd., Dongguan, China, comprised of N-P_2_O_5_-K_2_O, 15%–4%–6%, was applied at 900 kg ha^−1^ with 60% as a basal dose and 40% as topdressing. Water management practices were followed as adopted by local farmers.

### 4.2. Experimental Design

**Figure 9:**
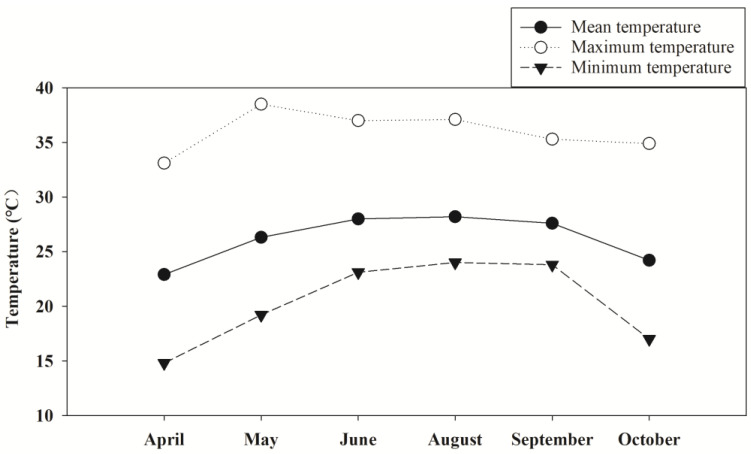
The monthly temperature during the field experiment.

Five nano-Se treatments were adopted in the present experiment. The nano-Se solution was foliar-applied: once at the end of the tillering (S1), heading (S2), and grain-filling (S3) stages or twice at the end of the tillering stage and the heading stage (S4), and at the end of the tillering stage and the grain-filling stage (S5). The treatment without Se application was taken as the control (CK). The treatments were arranged in a split block design with nano-Se treatment as the main factor and the cultivar as the secondary factor in triplicate in each cropping season with a net plot size of 21.60 m^2^. The nano-Se used was a simple substance Se produced by Green Huinong Biotechnology (Shenzhen) Co., Ltd., Shenzhen, China, with an average particle size of 13.767 nm. The Se content in the applied solution was 6.67 mg L^−1^ and one liter of diluted solution was for each plot. Water management in all plots was followed as adopted by Pan et al. [20]. After the transplanting, about 3 cm of water depth was kept until the end of the tillering stage. Subsequently, the water was drained for 7 days to control the infertile tillers. Then, a water level of 5–7 cm was kept at the following stages until 7 days before the harvest. In the early season, the sowing was conducted on 10 March. The transplanting was performed on 28 March. The harvest was performed on 10 July. In the late season, the sowing was conducted on 14 July. The transplanting was performed on 29 July. The harvest was performed on 23 October. The monthly temperature during the field experiment is shown in Figure 9.

### 4.3. Determination of Grain Yield, Yield-Related Traits, and Economic Profitability

At harvest, the rice grains were harvested from three sampling areas (1.00 m^2^) in each plot and then threshed by machine. The harvested grains were sun-dried and weighed to determine the grain yield. Thirty representative plants in each plot were investigated to calculate and record the effective panicle number per plant. Three representative plants in each plot were sampled to measure the grain number per panicle and the seed-setting rate (100 × filled grains number/total grains number) using a rice digital seed testing machine (YTS-5D, Red Star Yang Technology, Wuhan, China). The filled grains were weighed and used to calculate the 1000-grain weight.

### 4.4. Measurement of Grain Quality Parameters

The measurement of the grain quality was carried out according to the methods of Chen et al. [21]. The brown rice rate was measured by a rice huller (Hiroshima, Japan). The milled rice rate was measured by a Jingmi testing rice grader (Taizhou, China). The chalky rice rate and chalkiness degrees were measured by a scanner (MRS-9600TFUL, Shanghai Zhongjing Technology Co., Ltd., Shanghai, China) and a rice appearance quality analysis and detection system (Hangzhou Wanshen Testing Technology Co., Ltd., Hangzhou, China).

### 4.5. Determination of the Grain Se Content

For the determination of Se content, dry mature grains in each treatment were ground to the powder. The sample (0.5 g) in powder form was digested in a mixture of concentrated HNO_3_–HClO_4_ (4:1), and the Se content was determined using inductively coupled plasma atomic emission spectroscopy (ICP-AES) (SPS 1200 VR, Seiko Co., Ltd., Fukuoka, Japan) [22].

### 4.6. Determination of the Chlorophyll Content, Net Photosynthetic Rate, and Dry Matter Accumulation

At the grain-filling stage, the SPAD meter ‘SPAD-502’ (Konica Minolta, Tokyo, Japan) was used for precise, rapid, and non-destructive estimation of leaf chlorophyll content, and the chlorophyll content was expressed in SPAD units. Meanwhile, the net photosynthetic rate was measured with an LI-6400XT portable photosynthesis system (Licor, Lincoln, NE, USA) and expressed as μmol/m^2^/s. At the harvest, five representative plants were collected from each plot and oven-dried at 60 °C until a constant weight to measure the dry matter weight.

### 4.7. Determination of Superoxide Dismutase (SOD) and Catalase (CAT) Activity

At the grain-filling stage, flag leaves of rice plants were collected and used for determination of the activity of SOD and CAT. The determination of SOD activity was carried out using the nitro blue tetrazolium (NBT) method according to the methods of Mostofa et al. [23] and expressed as U/g/h. The determination of CAT activity was carried out according to the methods of Saha et al. [24] and expressed as mmol/(min·g).

### 4.8. Statistical Analysis

Experimental data in the present study were analyzed using the statistical software ‘Statistix8.1′ (Tallahassee, Florida, FL, USA) and Microsoft Office Excel 2016 (Microsoft, Washington, DC, USA). The differences among means were separated using the LSD test at the 5% probability level for each parameter in each cropping season.

## 5. Conclusions

Foliar application of nano-Se significantly increased the chlorophyll content, net photosynthetic rate, and dry matter accumulation of hybrid rice plants. Nano-Se application also enhanced the activity of antioxidant enzymes including SOD and CAT. The grain yield and yield-related traits such as the effective panicle number and seed-setting rate significantly increased under nano-Se applications and the highest grain yield was recorded in the S1 treatment which applied nano-Se once at the end of tillering. Furthermore, the foliar application of nano-Se remarkably increased the grain Se content of hybrid rice.

## Figures and Tables

**Table 1:** Effects of nano-Se on the yield and yield-related traits of hybrid rice.

Cropping Season	Cultivar	Treatment	Yield (t ha^−1^)	Effective Panicle Number per Plant	Grain Number per Panicle	Seed-Setting Rate (%)	1000-Grain Weight (g)
Early season							
	Jingnongsimiao						
		CK	4.43 ± 0.36 b	7.97 ± 0.24 b	134.91 ± 9.60 b	65.23 ± 3.20 b	19.44 ± 0.18 ab
		S1	6.16 ± 0.24 a	8.56 ± 0.06 a	172.07 ± 11.35 a	69.99 ± 1.68 b	18.63 ± 0.36 b
		S2	5.58 ± 0.28 a	8.33 ± 0.19 ab	146.5 ± 2.39 b	74.64 ± 3.67 ab	19.49 ± 1.19 ab
		S3	6.24 ± 0.14 a	8.19 ± 0.21 ab	147.59 ± 8.31 b	72.27 ± 3.69 ab	19.34 ± 0.34 ab
		S4	6.37 ± 0.55 a	8.40 ± 0.14 ab	144.5 ± 7.62 b	80.72 ± 4.54 a	19.19 ± 0.39 ab
		S5	5.49 ± 0.03 a	8.40 ± 0.06 ab	144.28 ± 2.31 b	68.03 ± 2.48 b	20.47 ± 0.47 a
	Wufengyou615						
		CK	5.86 ± 0.27 c	10.64 ± 0.32 b	108.04 ± 6.30 ab	75.64 ± 2.89 ab	20.43 ± 0.79 b
		S1	7.43 ± 0.51 ab	11.66 ± 0.34 a	119.19 ± 12.41 ab	73.84 ± 1.77 b	22.23 ± 0.47 a
		S2	7.07 ± 0.20 bc	11.17 ± 0.29 ab	102.19 ± 1.07 b	80.41 ± 1.63 ab	21.75 ± 0.37 ab
		S3	8.60 ± 0.69 a	11.23 ± 0.34 ab	161.79 ± 38.00 a	76.83 ± 5.01 ab	21.2 ± 0.64 ab
		S4	7.78 ± 0.29 ab	11.37 ± 0.35 ab	142.42 ± 11.51 ab	81.43 ± 1.50 ab	22.40 ± 0.51 a
		S5	8.41 ± 0.20 a	11.33 ± 0.27 ab	127.59 ± 10.49 ab	84.67 ± 5.31 a	22.75 ± 0.28 a
Late season							
	Jingnongsimiao						
		CK	4.06 ± 0.11 b	8.27 ± 0.07 b	135.03 ± 16.23 a	63.72 ± 1.01 c	20.97 ± 0.45 ab
		S1	5.19 ± 0.23 a	9.40 ± 0.12 a	138.01 ± 11.55 a	78.38 ± 2.07 a	21.64 ± 0.20 ab
		S2	4.78 ± 0.24 a	9.27 ± 0.07 a	140.19 ± 8.91 a	69.73 ± 3.13 bc	22.14 ± 0.56 a
		S3	4.66 ± 0.09 a	9.13 ± 0.18 a	121.66 ± 11.82 a	64.55 ± 1.84 c	21.55 ± 0.34 ab
		S4	4.91 ± 0.20 a	9.53 ± 0.18 a	121.6 ± 8.13 a	76.44 ± 3.67 ab	20.50 ± 0.28 b
		S5	4.97 ± 0.21 a	9.33 ± 0.13 a	131.03 ± 0.83 a	71.89 ± 0.59 ab	21.72 ± 0.54 ab
	Wufengyou615						
		CK	4.36 ± 0.26 c	10.2 ± 0.12 c	148.11 ± 7.62 ab	81.41 ± 0.92 b	22.79 ± 0.59 b
		S1	6.13 ± 0.37 a	11.33 ± 0.27 a	127.22 ± 6.44 b	88.40 ± 1.93 a	22.83 ± 0.37 b
		S2	5.06 ± 0.05 bc	10.93 ± 0.24 ab	130.69 ± 10.70 ab	78.38 ± 2.01 b	22.52 ± 0.11 b
		S3	6.10 ± 0.44 a	10.20 ± 0.12 c	130.16 ± 5.44 ab	88.17 ± 0.36 a	22.97 ± 0.12 b
		S4	5.92 ± 0.24 ab	10.47 ± 0.18 bc	140.59 ± 4.08 ab	88.51 ± 0.20 a	24.28 ± 0.57 a
		S5	6.10 ± 0.19 a	10.67 ± 0.18 bc	161.77 ± 20.29 a	89.76 ± 3.98 a	23.52 ± 0.30 ab
Analysis of variance							
Season			**	ns	ns	ns	**
Cultivar			**	**	ns	**	**
Season × Cultivar			**	**	ns	**	ns
Nano-Se			**	**	ns	**	*
Season × Nano-Se			ns	ns	ns	ns	ns
Cultivar × Nano-Se			ns	ns	ns	ns	**
Season × Cultivar × Nano-Se			ns	*	ns	ns	ns

Note: Values sharing a common letter within a column do not differ significantly at *p* ≤ 0.05 in each cropping season according to the LSD test. For analysis of variance, * and ** represent a significant difference at *p* < 0.05 and *p* < 0.01, respectively; ns represents a non-significant. The nano-Se solution was foliar-applied: once at the end of the tillering (S1), heading (S2), and grain-filling (S3) stages or twice at the end of the tillering stage and the heading stage (S4), and at the end of the tillering stage and the grain-filling stage (S5). The treatment without Se application was taken as the control (CK), the same as below.

**Table 2:** Effects of nano-Se on grain quality parameters of hybrid rice.

Cropping Season	Cultivar	Treatment	Brown Rice Rate (%)	Milled Rice Rate (%)	Head Rice Rate (%)	Chalky Rice Rate (%)	Chalkiness Degree (%)
Early season							
	Jingnongsimiao						
		CK	95.92 ± 0.65 a	86.22 ± 0.31 ab	78.35 ± 0.33 a	9.07 ± 0.24 a	7.03 ± 0.55 a
		S1	96.53 ± 0.54 a	85.18 ± 0.75 ab	78.69 ± 0.36 a	6.97 ± 2.23 a	5.12 ± 2.25 a
		S2	97.82 ± 0.60 a	84.86 ± 0.81 b	78.77 ± 0.52 a	9.40 ± 0.40 a	7.58 ± 0.06 a
		S3	97.80 ± 0.51 a	86.19 ± 0.07 ab	78.73 ± 0.33 a	9.20 ± 0.32 a	7.41 ± 0.32 a
		S4	96.65 ± 1.32 a	86.30 ± 0.23 ab	77.75 ± 0.17 a	9.20 ± 0.40 a	7.73 ± 0.17 a
		S5	98.03 ± 0.08 a	86.40 ± 0.34 a	78.39 ± 0.12 a	7.43 ± 19.80 a	4.48 ± 1.79 a
	Wufengyou615						
		CK	96.29 ± 0.51 a	84.23 ± 0.09 ab	78.31 ± 0.55 b	5.43 ± 0.90 ab	1.97 ± 0.45 a
		S1	96.39 ± 0.42 a	83.68 ± 0.43 b	80.42 ± 0.17 a	6.77 ± 0.95 a	2.48 ± 0.63 a
		S2	96.17 ± 0.06 a	83.59 ± 0.45 b	78.14 ± 1.15 b	5.87 ± 1.12 ab	2.01 ± 0.44 a
		S3	94.81 ± 0.89 a	84.02 ± 0.06 ab	78.78 ± 0.12 ab	6.10 ± 0.50 ab	2.19 ± 0.27 a
		S4	95.25 ± 1.60 a	84.72 ± 0.39 a	78.45 ± 0.32 b	4.40 ± 0.31 b	1.43 ± 0.05 a
		S5	94.39 ± 1.83 a	84.29 ± 0.22 ab	79.04 ± 0.16 ab	5.93 ± 0.45 ab	2.14 ± 0.22 a
Late season							
	Jingnongsimiao						
		CK	98.67 ± 0.13 a	94.1 ± 0.05 ab	70.90 ± 0.16 b	1.00 ± 0.00 c	0.28 ± 0.10 a
		S1	97.94 ± 0.24 b	94.15 ± 0.05 a	71.69 ± 0.61 ab	2.33 ± 0.67 abc	0.54 ± 0.35 a
		S2	98.30 ± 0.28 ab	93.78 ± 0.05 b	72.39 ± 0.53 ab	3.33 ± 0.67 a	0.79 ± 0.48 a
		S3	98.60 ± 0.32 ab	94.17 ± 0.24 a	70.68 ± 1.14 b	1.33 ± 0.33 bc	0.32 ± 0.21 a
		S4	98.44 ± 0.25 ab	93.9 ± 0.05 ab	73.10 ± 0.46 a	3.00 ± 0.58 ab	0.68 ± 0.06 a
		S5	98.54 ± 0.03 ab	93.91 ± 0.03 ab	72.52 ± 0.43 ab	3.33 ± 0.88 a	0.45 ± 0.08 a
	Wufengyou615						
		CK	98.97 ± 0.13 ab	92.15 ± 0.21 ab	74.59 ± 0.29 c	3.00 ± 0.58 a	0.58 ± 0.10 a
		S1	99.37 ± 0.14 a	92.30 ± 0.07 ab	75.99 ± 0.48 bc	2.00 ± 0.58 a	0.39 ± 0.25 a
		S2	99.19 ± 0.11 a	92.34 ± 0.08 ab	76.47 ± 0.27 ab	2.67 ± 0.33 a	0.34 ± 0.05 a
		S3	99.26 ± 0.16 a	92.27 ± 0.10 ab	76.58 ± 0.85 ab	3.00 ± 1.15 a	0.46 ± 0.22 a
		S4	98.68 ± 0.18 b	92.44 ± 0.02 a	77.79 ± 0.54 a	3.33 ± 0.67 a	0.95 ± 0.4 a
		S5	99.34 ± 0.11 a	92.07 ± 0.10 b	76.73 ± 0.30 ab	3.00 ± 0.00 a	0.52 ± 0.13 a
Analysis of variance							
Season			**	**	*	**	**
Cultivar			**	**	ns	*	**
Season × Cultivar			**	ns	*	**	**
Nano-Se			**	*	ns	ns	ns
Season × Nano-Se			**	*	ns	ns	ns
Cultivar × Nano-Se			ns	ns	ns	ns	ns
Season × Cultivar × Nano-Se			ns	ns	ns	ns	ns

## Data Availability

The original contributions presented in the study are included in the article, and further inquiries can be directed to the corresponding authors.
